# The Function of Different Subunits of the Molecular Chaperone CCT in the Microsporidium *Nosema bombycis*: NbCCTζ Interacts with NbCCTα

**DOI:** 10.3390/jof10030229

**Published:** 2024-03-20

**Authors:** Sheng Xu, Ying Chen, Jingru Qi, Runpeng Wang, Erjun Wei, Qiang Wang, Yiling Zhang, Xudong Tang, Zhongyuan Shen

**Affiliations:** 1 College of Biotechnology, Jiangsu University of Science and Technology, Zhenjiang 212100, China; xu18609659561@163.com (S.X.); 17853577156@163.com (Y.C.); jingru.qi@biortus.bio (J.Q.); qb1999517@126.com (R.W.); wejjust@163.com (E.W.); wqiang523@126.com (Q.W.); zhangyiling008@126.com (Y.Z.); xudongt@just.edu.cn (X.T.); 2 Sericulture Research Institute, Chinese Academy of Agricultural Sciences, Zhenjiang 212100, China

**Keywords:** microsporidia, chaperonin, CCTζ, coordinating function, immunolocalization

## Abstract

Chaperonin containing tailless complex polypeptide 1 (CCT) is a molecular chaperone protein that consists of eight completely different subunits and assists in the folding of newly synthesized peptides. The zeta subunit of CCT is a regulatory factor for the folding and assembly of cytoskeletal proteins as individuals or complexes. In this study, the zeta subunit of *Nosema bombycis* (NbCCTζ) is identified for the first time. The complete ORF of the *NbCCTζ* gene is 1533 bp in length and encodes a 510 amino acid polypeptide. IFA results indicate that NbCCTζ is colocalized with actin and β-tubulin in the cytoplasm during the proliferative phase and that NbCCTζ is completely colocalized with NbCCTα in the cytoplasm of *N. bombycis* throughout the entire life cycle. Furthermore, the yeast two-hybrid assay revealed that the NbCCTζ interacts with NbCCTα. The transcriptional level of *NbCCTζ* is significantly downregulated by knocking down the *NbCCTα* gene, while the transcriptional level of *NbCCTα* is downregulated after knocking down the *NbCCTζ* gene. These results suggest that NbCCTζ may play a vital role in the proliferation of *N. bombycis* by coordinating with NbCCTα.

## 1. Introduction

Microsporidia are obligate intracellular parasites that are capable of infecting diverse vertebrates and invertebrates including humans [1]. So far, 220 genera and 1700 species of microsporidia have been identified [2]. *Nosema bombycis* is the pathogen causing pebrine disease in silkworms and is transmitted both horizontally and vertically to silkworms and leads to considerable economic losses in the sericulture industry [3].

The synthesis and folding of nascent peptides occur throughout the entire life cycle of microsporidia. The chaperonin-containing tailless complex polypeptide 1 (CCT), a molecular chaperone protein known as the T-complex 1, plays a crucial role in the correct folding of proteins [4]. Each loop of CCT has eight different subunits (CCT1–CCT8, also known as CCTα–CCTθ) with three characteristic domains: the apical domain, the equatorial domain, and the intermediate domain. The apical domain comprises the loop that forms the lid, the equatorial domain encompasses the ATP binding site, and the intermediate domain contains the conserved aspartic acid essential for ATP hydrolysis [5,6,7]. A growing number of studies show that about 10–15% of newly synthesized proteins interact with CCT, including numerous essential structural and regulatory proteins such as the cell cycle regulator CDC20, the cancer-related VHL tumor suppressor, and WD40-repeat proteins [7,8,9]. Meanwhile, CCT is also involved in many fundamental cellular processes, including cell division [10], metabolism, transcription and translation [11], cellular trafficking, and signal transduction [12].

The cellular functions of the eukaryotic CCT chaperone were first associated only with the folding of the cytoskeletal protein tubulin and actin [6]; however, the sequences of the substrate-binding domains at the top of the eight subunits differ remarkably, resulting in different types of substrate binding. Therefore, different subunits may have different functions as well [13,14]. CCTα is necessary for ciliate assembly and the maintenance of axial filament structure [15]. CCTβ and CCTδ bind to actin, while tubulin interacts with all CCTs [6]. CCTθ is associated with the migration of tumor cells, and ectopic expression leads to the high aggressiveness of esophageal squamous cell carcinoma (ESCC) [16,17], whereas CCTγ inhibits the migration of cancer cells [18,19]. CCTζ is a regulatory factor for cytoskeleton assembly and cell cycle regulation [20,21].

In this study, the NbCCTζ (the CCTζ of *N. bombycis*) was identified for the first time. NbCCTζ is colocalized with Nb-actin and Nbβ-tubulin in the cytoplasm of proliferating *N. bombycis*. Moreover, NbCCTζ interacts with NbCCTα and is colocalized with NbCCTα. The knockdown of *NbCCTζ* resulted in the downregulated expression of the *NbCCTα* gene, while the expressional level of the *NbCCTζ* gene was also affected by the knockdown of the *NbCCTα* gene. Our data suggest that the NbCCTζmay play a vital role in the proliferation of *N. bombycis*.

## 2. Materials and Methods

### 2.1. Parasite and Host

The NbCCTα antibody, Nbβ-tubulin antibody, Nb-actin antibody, and *N. bombycis* were provided by the Sericultural Research Institute of the Chinese Academy of Agricultural Sciences.

### 2.2. Cloning and Bioinformatic Analysis of NbCCTζ

The purified spore suspension of *N. bombycis* (10^9^ sopres/mL) was ground for 1 min with a bead grinder and then cooled on ice for 5 min, repeated 6 times. The genomic DNA was extracted with a fungal genomic DNA extraction kit and stored at −20 °C following concentration measurement.

According to the partial sequences of *NbCCTζ* on the NCBI database as well as the homologous sequences of the *CCTζ* of other microsporidian species, the specific forward primer 5′-CCGGAGCTCATGCAATCCACTCAATCCGAC-3′ and reverse primer 5′-CCCTCGAGTTATTGTTCAATCTTCTCTTCCTTA-3′ were designed and synthesized (Sangon Biotech, Shanghai, China). PCR amplification was performed using the *N. bombycis* genomic DNA as a template comprising 25 μL of PrimeSTAR HS DNA Polymerase (Takara Biotechnology, Dalian, China), 2 μL per primer, 2 μL of genomic DNA, and 19 μL of ddH2O. The following parameters were applied in the amplification system: predenaturation at 95 °C for 5 min and PCR amplification at 98 °C for 10 s, 55 °C for 15 s, and 72 °C for 90 s, totaling 35 cycles. The amplified product was detected on 1% agarose gel and was purified using an Axyprep DNA gel extraction kit (AP-GX-4, Axygen Bioscience, Union City, CA, USA). Then, the product with polyA tail was ligated to the pMD19-T vector overnight at 16 °C. The pMD19-T-NbCCTζ recombinant plasmid was transformed into the TOP10 competent cells. Positive clones were confirmed through blue-white screening, and the recombinant plasmids were sequenced by Sangon Biotech (Shanghai, China).

Multiple sequence alignment was performed by using DNAMAN8 software (DNAMAN8.0 for Windows). The isoelectric point and relative molecular mass of protein were predicted on Compute_Pi/Mw (https://web.expasy.org/compute_pi/, accessed on 23 January 2023). The transmembrane domain was predicted with TMHMM Server v (http://www.cbs.dtu.dk/services/TMHMM/, accessed on 23 January 2023). N-glycosylation modification was predicted with NetNGlyc 1.0 (http://www.cbs.dtu.dk/services/NetNGlyc/, accessed on 23 January 2023). O-glycosylation was predicted with YinOYang 1.2 (https://services.healthtech.dtu.dk/service.php?YinOYang-1.2, accessed on 23 January 2023). Phosphorylation was predicted by using NetPhos 3.1 (https://services.healthtech.dtu.dk/service.php?NetPhos-3.1, accessed on 24 January 2023). The signal peptide was predicted by SignalP5.0 (http://www.cbs.dtu.dk/services/SignalP/, accessed on 24 January 2023). The secondary structure was predicted on PSIPRED (http://bioinf.cs.ucl.ac.uk/psipred/, accessed on 24 January 2023). The subcellular localization of NbCCTζ was predicted using Cell-PLoc2.0 (http://www.csbio.sjtu.edu.cn/bioinf/Cell-PLoc-2/, accessed on 24 January 2023).

### 2.3. Expression and Purification of NbCCTζ Recombinant Protein

By analyzing the sequence of hydrophobicity, homology, antigenicity, hydrophilicity, and the possibility of protein expression in a prokaryotic system, the sequence region 168–390 with a length of 223 amino acids was selected for antibody preparation. The target sequence was amplified with the specific primers F-*BamH* I (5′-TCAGGATCCGTTAAAGCTATAAAAAATATCTC-3′) and R-*Xho* I (5′-GATCTCGAGTTTAAGAGAGGCTCTAAT-3′), and then the pET28a-NbCCTζ-233 plasmid was generated by inserting the *NbCCTζ*-233 gene into *BamH* I and *Xho* I-digested pET28a plasmid. The NbCCTζ-233 recombinant protein was expressed and purified according to a previous report [22].

### 2.4. Preparation of NbCCTζ Polyclonal Antibody and Western Blot

The purified protein obtained above was used to prepare polyclonal antibodies. Briefly, New Zealand rabbits were immunized with equal amounts of recombinant protein and incomplete Freund’s adjuvant every 15 days, and the serum was collected on the 53rd day. Polyclonal antibodies were obtained by using antigen affinity column purification, and the preimmune serum was collected as a negative control.

The total protein of *N. bombycis* was extracted by breaking the spores with acid-washed glass beads. NbCCTζ was detected in the total protein of *N. bombycis* using the NbCCTζ antibody.

### 2.5. Immunolocalization of NbCCTζ in N. bombycis

Mature spores of *N. bombycis* were germinated in 0.1 M KOH at 27 °C for 0.5 h. The BmN cells were inoculated with the germinating spores in six-well plates. The colocalization of NbCCTζ with Nbβ-tubulin, Nb-actin, or NbCCTα was investigated according to our previous work [23].

### 2.6. RNAi

Small interfering RNA (siRNA) was designed and synthesized by Sangong Biotech (Shanghai, China). Fresh mulberry leaves were coated with 10^8^ spores/mL of microsporidia. The silkworm larvae of the fifth instar were fed on the above-obtained mulberry leaves. Following 6 h of feeding, 3 μL of siRNA (Table 1) was injected into each larva. The midguts from three independent silkworm samples were collected at 24 h, 48 h, 72 h, and 96 h post-siRNA injection and stored at −80 °C.

The specimens were lysed with 1 mL of RNAiso plus lysate (9108Q, Takara, Dalian, China), and the total RNA was extracted with a Mini Best universal RNA extraction kit (9767, Takara Biotechnology, Dalian, China). cDNA was synthesized with a PrimeScript RT master mix (RR036B, Takara Biotechnology, Dalian, China) and was used as the template. RT-qPCR was performed by using the TB green premix Ex Taq II (Tli RNase H Plus) kit (RR820A, Takara Biotechnology, Dalian, China). The primer sequences are listed in Table 1. The transcriptional level was calculated by using the 2^−∆∆ct^ method with three replicates. The multiple *t*-test was conducted using GraphPad Prism 8.0 (GraphPad Software, San Diego, CA, USA).

### 2.7. Yeast Two-Hybrid Assay

The *NbCCTα* gene was amplified using the genomic DNA of *N. bombycis* with specific primers (F-*EcoR* I: 5′-CATGGAGGCCGAATTCATGTTCAACGAAATCTCTACTAACA-3′, R-*Not* I: 5′-GCTAGTTATGCGGCCTTATTTCCTGTTTGGCATTATTATT-3′). The *NbCCTζ* gene was amplified using specific primers (F-*EcoR* I: 5′-GGAGGCCAGTGAATTCATGCAATCCACTCAATCCGA-3′, R-*Xho* I: 5′-TCATCTGCAGCTCGATTATTGTTCAATCTTCTCTTCCTT-3′). The *NbCCTα* gene was ligated into the *EcoR* I and *Not* I-digested yeast two-hybrid bait vector pGBKT7-BK, whereas the *NbCCTζ* gene was ligated into *EcoR* I and *Xho* I-digested prey pGADT7-AD using an In-Fusion HD Cloning Kit (638909, Takara, Kusatsu, Japan), respectively. The bait recombinant vector and the prey recombinant vector were cotransformed into Y2H Gold yeast cells (YC1002, Weidi Biotech, Shanghai, China). The cotransformed cells were cultured on SD/-Leu/-Trp plates (630489, TaKaRa, Kusatsu, Japan) at 28 °C. The single colony on SD/-Leu/-Trp plates was chosen and cultured on SD/-Ade/-His/-Leu/-Trp/X-a-gal plates (630462, TaKaRa, Kusatsu, Japan) at 28 °C.

## 3. Results

### 3.1. Cloning and Expression of NbCCTζ and Western Blot

**Figure 1:**
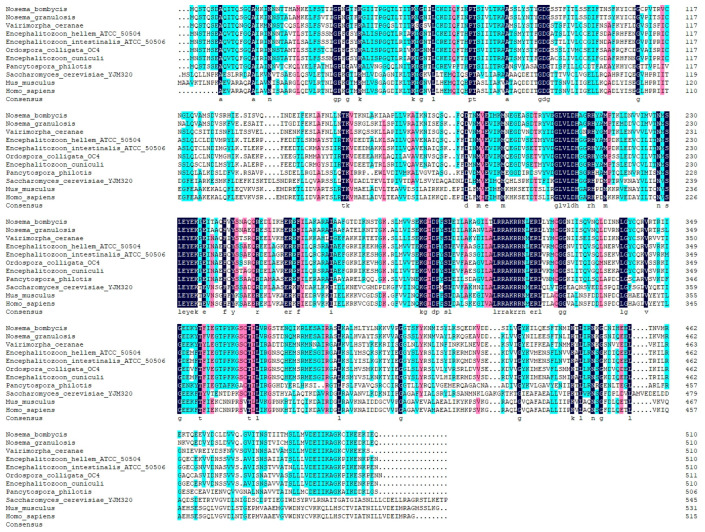
Multiple sequence alignment between CCTζ from *Nosema bombycis* and other eukaryotes.

The complete nucleotide sequence of the *NbCCTζ* gene (NCBI accession number: PP069767.1) was obtained by PCR amplification and sequencing. The results showed that the ORF of the *NbCCTζ* gene was 1533 bp and that it encodes a polypeptide of 510 amino acids (Figure S1). The bioinformatic analysis showed that the predicted molecular weight is 57 kDa and that there is no signal peptide and no transmembrane domain. The prediction of phosphorylation sites and glycosylation sites indicated that the NbCCTζmay have 32 phosphorylation sites and 2 N-glycosylation sites. The secondary structure analysis showed that the NbCCTζ gene is mainly composed of helix and coil. Multiple sequence alignment indicated that NbCCTζ shares a certain degree of homology with that of other microsporidia, especially *Nosema granulosis* (79.80%) and *Vairimorpha ceranae* (62.28%), but has a low degree of homology with that of other eukaryotes, such as *Saccharomyces cerevisiae* (30.64%) (Figure 1), indicating that CCTζ is not conserved in the eukaryote.

**Figure 2:**
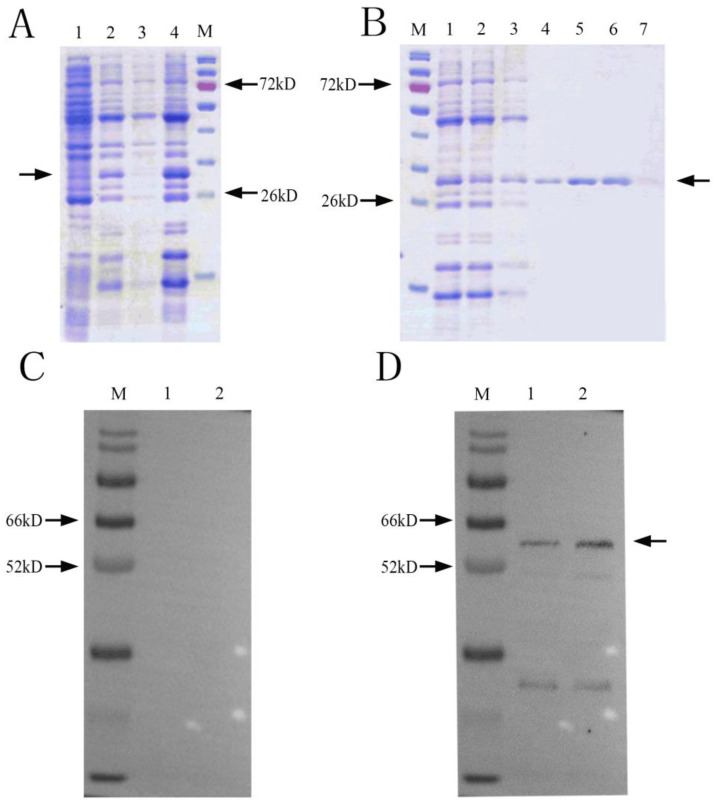
Recombinant protein expression and purification and Western blot analysis: (**A**) SDS-PAGE of expressed recombinant protein M: protein marker; 1: NbCCTζ-233 total cell lysate (uninduced); 2: NbCCTζ-233 total cell lysate (4 h after induction with 0.5 mM IPTG, 37 °C); 3: ultrasonic supernatant; 4: ultrasonic precipitation; Arrow indicates NbCCTζ-233 protein. (**B**) protein purification M: protein marker; 1: ultrasonic precipitation with urea solution (sample to be purified); 2: flowthrough; 3: 10 mM imidazole elution; 4: 20 mM imidazole elution; 5: 50 mM imidazole elution 6: 200 mM imidazole elution; 7: 500 mM imidazole elution; Arrow indicates NbCCTζ-233 protein (**C**) preimmune serum M: protein marker; 1 and 2: total protein of *N. bombycis*; (**D**) Western blot of NbCCTζ M: protein marker; 1 and 2: total protein of *N. bombycis*. Arrow indicates NbCCTζ protein.

SDS-PAGE results showed that there was an obvious band at about 29 kDa (Figure 2A). After ultrasonication, there was a clear protein band at 29 kDa in the ultrasonic precipitate, indicating that the NbCCTζ-233 was mainly expressed in the inclusion bodies. Ni-NTA purification results showed that the NbCCTζ-233 was well bound to the NTA column under denaturing conditions, and the target protein could be obtained by elution with 50 mM or 200 mM imidazole (Figure 2B). Western blot using preimmune serum as the negative control showed a specific band at about 57 kDa in the total proteins of *N. bombycis* (Figure 2C,D). These results indicate that the polyclonal antibody undergoes a specific antigen–antibody reaction.

### 3.2. Subcellular Localization of NbCCTζ in N. bombycis

**Figure 3:**
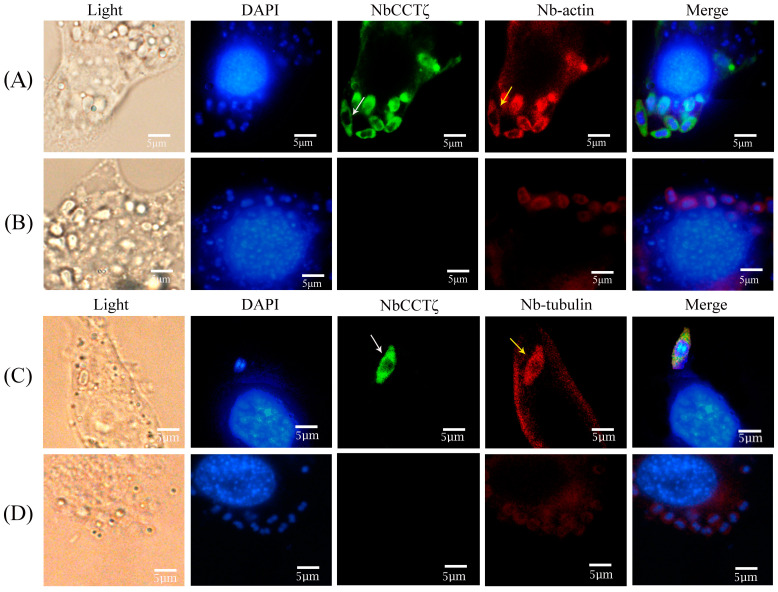
Colocalization analysis of NbCCTζ with Nb-actin and Nbβ-tubulin in the proliferative phase of *N. bombycis*: (**A**,**C**) proliferative phase; (**B**,**D**) preimmune serum. The white arrow indicates NbCCTζ; the yellow arrow indicates the Nb-actin or Nbβ-tubulin. The white arrows indicated the green fluorescent signals (NbCCTζ) and the yellow arrows indicated the red fluorescent signals (Nb-actin or Nbβ-tubulin).

An IFA (immunofluorescence assay) was performed to investigate the subcellular localization of NbCCTζ in the proliferative phase of *N. bombycis*. The NbCCTζ antibody was coupled with Alexa Fluor 488 (green), while the Nb-actin antibody or the Nbβ-tubulin antibody was coupled with Cy5 (red), and the nucleus was stained with DAPI (blue). IFA results show that the NbCCTζ genes are mainly distributed in the cytoplasm and completely overlap with Nb-actin (Figure 3A) or Nbβ-tubulin (Figure 3C) during the proliferative phase.

### 3.3. NbCCTζ Interacts with NbCCTα in Yeast Two-Hybrid Assay

**Figure 4:**
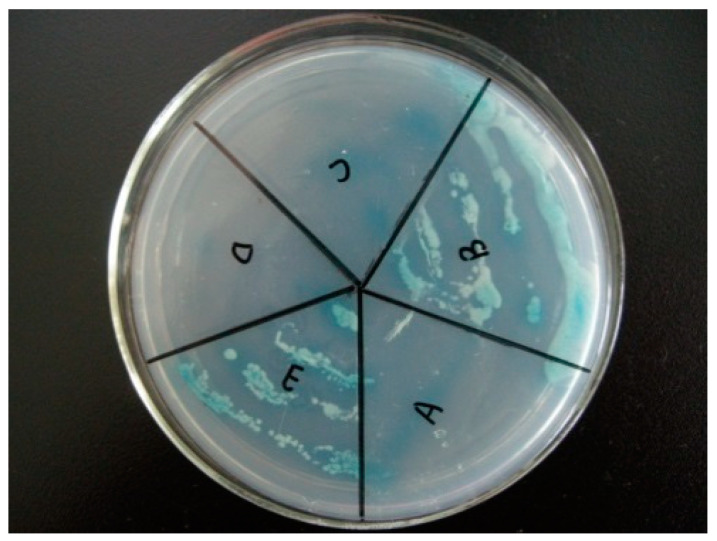
Validation of yeast two-hybrid positive plasmids: (A) negative control (pGBKT7-lam and pGADT7-T); (B) positive control (pGBKT7-p53 and pGADT7-T); (C) pGADT7 and pGBKT7-NbCCTα; (D) pGBKT7 and pGADT7-NbCCTζ; (E) pGBKT7-NbCCTα and pGADT7-NbCCTζ.

The yeast with NbCCTα-PGBKT7 and NbCCTζ-PGADT7 plasmids (Figure 4E) and the positive control (Figure 4B) strains with pGBKT7-p53 and pGADT7-T plasmids could still grow in a four-deficient medium (SD/-Leu/-Trp/-His/-Ade), while the negative control strains with pGBKT7-lam and pGADT7-T (Figure 4A) could not grow on this medium, indicating that NbCCTζ has a strong interaction with NbCCTα.

### 3.4. Colocalization of NbCCTζ and NbCCTα

**Figure 5:**
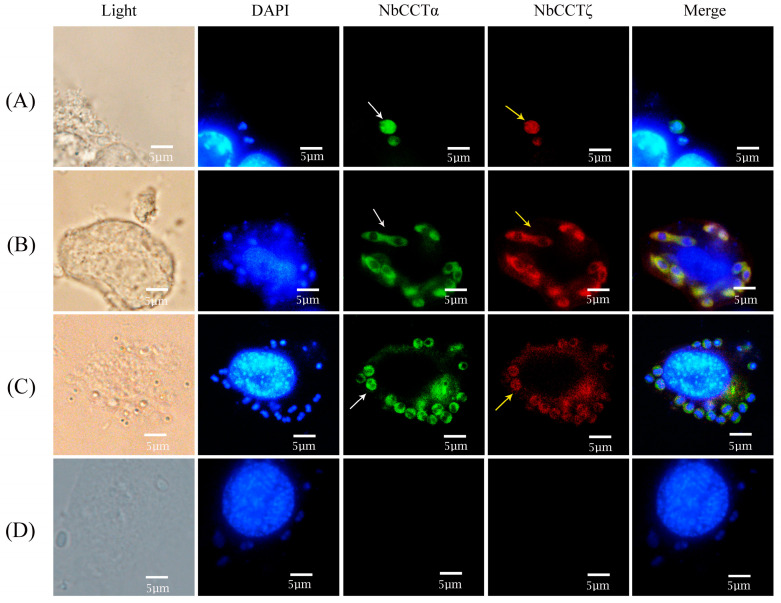
Colocalization analysis of NbCCTζ and NbCCTα in the intracellular stage of *N. bombycis*: (**A**) sporoplasm; (**B**) meront; (**C**) sporoblast; (**D**) negative control. The white arrows indicated the green fluorescent signals (NbCCTζ) and the yellow arrows indicated the red fluorescent signals (NbCCTα).

To investigate the colocalization of NbCCTζ with other subunits in the intracellular phase of *N. bombycis*, NbCCTα and NbCCTζ were selected for colocalization using the IFA. NbCCTα and NbCCTζ are labeled with green fluorescence and red fluorescence, respectively. In sporoplasm, NbCCTα and NbCCTζ were distributed in the whole cell (Figure 5A). In the proliferative phase (Figure 5B) and the sporogenic phase (Figure 5C), NbCCTα and NbCCTζ were mainly distributed in the cytoplasm. These results indicate that the NbCCTα and NbCCTζare colocalized throughout the entire life cycle of *N. bombycis*.

### 3.5. Knockdown of NbCCTα Downregulated the Expression of NbCCTζ, and the Knockdown of NbCCTζ Downregulated the Expression of NbCCTα

**Figure 6:**
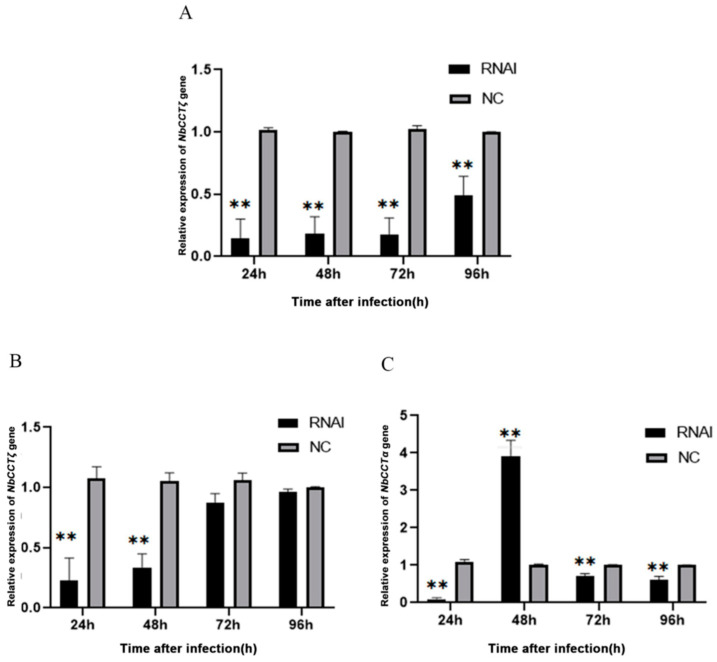
Transcriptional level of *NbCCTζ* and *NbCCTα* after RNAi: (**A**) the relative expression of the *NbCCTζ* gene after RNAi of *NbCCTα*. Error bars represent the standard deviation of three independent replicates (*n* = 3, mean ± SE, ** *p* < 0.01); (**B**) the relative expression of the *NbCCTζ* gene after RNAi of *NbCCTζ*. Error bars represent the standard deviation of three independent replicates (*n* = 3, mean ± SE, ** *p* < 0.01); (**C**) the relative expression of the *NbCCTα* gene after the RNAi of *NbCCTζ*. Error bars represent the standard deviation of three independent replicates (*n* = 3, mean ± SE, ** *p* < 0.01).

Our previous work has proved that RNAi can downregulate the expression of NbCCTα [24]. In this study, after the knockdown of *NbCCTα* or *NbCCTζ*, the relative expression of *NbCCTζ* or *NbCCTα* was detected by RT-qPCR using *NbssrRNA* as a reference gene. The transcriptional level of the *NbCCTζ* gene was extremely significantly downregulated at 24 h, 48 h, 72 h, and 96 h by the RNAi of *NbCCTα* (Figure 6A). After the RNAi of *NbCCTζ*, the transcriptional level of *NbCCTζ* was significantly downregulated at 24 h and 48 h (Figure 6B), while the transcriptional level of *NbCCTα* was extremely significantly downregulated at 24 h, 72 h, and 96 h (Figure 6C). These results indicate that NbCCTζ may functionally cooperate with NbCCTα.

### 3.6. Knocking down NbCCTζ Suppressed the Proliferation of N. bombycis

**Figure 7:**
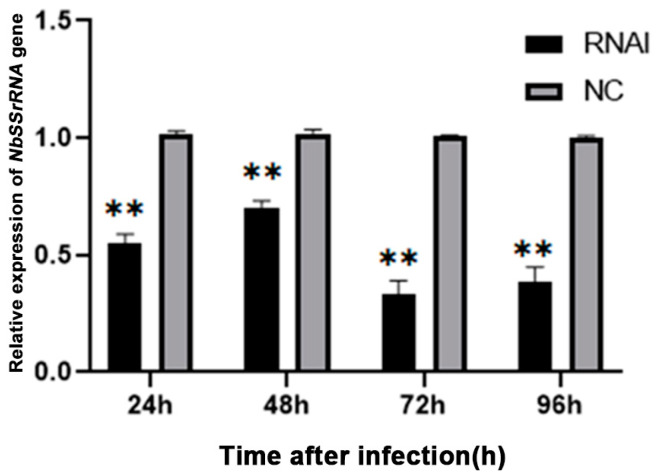
Relative expression of *NbssrRNA* gene after RNAi of *NbCCTζ*. Error bars represent the standard deviation of three independent replicates (*n* = 3, mean ± SE, ** *p* < 0.01).

The relative expression of the *NbCCTζ* gene was detected at all time points after infection and exhibited a gradual decline during the lifecycle of *N. bombycis* (Figure S2). To explore the effect of knocking down the *NbCCTζ* gene on the proliferation of *N. bombycis*, the transcriptional level of *NbssrRNA* was determined, which reflected the proliferation of *N. bombycis*. The effect of RNAi was analyzed by qPCR using *BmGAPDH* (glyceraldehyde-3-phosphate dehydrogenase of *Bombyx mori*) as a reference gene. The transcriptional level of *NbssrRNA* was significantly downregulated by the RNAi of *NbCCTζ* at 24 h, 48 h, 72 h, and 96 h post-infection (Figure 7). These results suggest that *NbCCTζ* plays an important role in the proliferation of *N. bombycis*.

## 4. Discussion

CCT complexes are a class of molecular chaperones with highly homologous sequences and are involved in many basic cellular processes in organisms; they were first discovered in mouse spermatogonia by Silver [25]. They also help proteins properly fold, maintain protein conformation, and are critical for cell survival and growth [26]. Microsporidia have reduced numbers of chaperone proteins outside the CCT [27]; however, to date, no CCT complex has been found in microsporidia using cryo-EM [28,29].

Some CCT subunits, such as CCTα, CCTγ, and CCTζ, play an important role in the function of microtubule-associated proteins [30]. The C-terminal region of tubulin interacts with CCTζ [31]. In this study, our findings demonstrated that the NbCCTζ gene exhibited limited homology with eukaryotic counterparts while displaying a certain degree of similarity to other microsporidia. Nakjang S et al. found that the proteins that are conserved across all microsporidian genomes are likely to be particularly important for the maintenance of their parasitic lifestyle [32]. Our IFA results show that the NbCCTζis mainly distributed in the cytoplasm and is colocalized with Nb-actin and Nbβ-tubulin in the proliferative phase. During the proliferative phase, the cytoskeletal proteins such as actin and tubulin are actively synthesized to enable cell division, as well as the intracellular transport of the components of the spore wall that starts forming at this stage [33]. These lines of evidence are similar to our previous findings on NbCCTα [24] and indicate that NbCCTζ may have a function like NbCCTα in the folding of tubulin and actin.

CCT consists of two heterogeneous eight-membered rings stacked back to back, forming a structure that encloses a folding cavity [5]. However, the arrangement of CCT subunits may be different in eukaryotes [34,35], and CCT subunits do not always assemble into a single form of hetero-oligomeric complex; instead, they confer independent functions in the cell [36]. Cong Y et al. reported that CCTζ interacts with CCTα in the 4.0-A° single-particle cryo-EM-derived structure of bovine testis CCT [34]. The present study shows that NbCCTζ interacts with NbCCTα, and NbCCTζ is colocated with NbCCTα throughout the entire life cycle of *N. bombycis*. The transcriptional level of *NbCCTζ* was significantly downregulated by knocking down the *NbCCTα* gene. The transcriptional level of *NbCCTα* was downregulated after the knockdown of the *NbCCTζ* gene at 24 h, 72 h, and 96 h, whereas it was significantly upregulated at 48 h; moreover, the proliferation of *N. bombycis* was significantly inhibited, indicating that the NbCCTζplays a crucial role in the proliferation of *N. bombycis*. Our previous studies have revealed that NbCCTα is related to cell division, and NbCCTδ plays an efficient role in keeping the integrity of the cytoskeleton [24,37]. Combining the results of this study with those of our previous work, we summarize our knowledge of the expression pattern and subcellular localization of CCTζ, CCTδ, and CCTα in *N. bombycis* in Table S1, which may help to perform further research on the function and relationship of CCT subunits in microsporidia. Taken together, these lines of evidence suggest that the subunits of CCT not only perform independent functions but also cooperate to assist the modification of protein.

## Figures and Tables

**Table 1:** Primer sequences for RNAi and RT-qPCR.

Gene		Sequences
*NbCCTζ* (RNAi)	Sense Antisense	GCUAUUAGAGCCUCUCUUATT (5′-3′) UAAGAGAGGCUCUAAUAGCTT (5′-3′)
Negative control (RNAi)	Sense Antisense	UUCUCCGAACGUGUCACGUTT (5′-3′) ACGUGACACGUUCGGAGAATT (5′-3′)
*NbCCTζ* (RT-qPCR)	Forward Reverse	AGCCAGAGCTATTGCTGCTTTTGG (5′-3′) GAATACCAGCCTTGGCAAGGATTTC (5′-3′)
*NbCCTα* (RT-qPCR)	Forward Reverse	GGTGGGGCTATTGAAGTCGC (5′-3′) CCAAACCCGCATTTATTGCCA (5′-3′)
*BmGAPDH* (RT-qPCR)	Forward Reverse	TTCATGCCACAACTGCTACA (5′-3′) AGTCAGCTTGCCATTAAGAG (5′-3′)
*SSrRNA* (RT-qPCR)	Forward Reverse	TCGAGTGCCAGCAGCCGCGG (5′-3′) CGATCCTCTAGCTTACGTCC (5′-3′)

## Data Availability

Data are contained within the article and Supplementary Materials.

## Supplementary Materials

The following supporting information can be downloaded at: https://www.mdpi.com/article/10.3390/jof10030229/s1, Figure S1: The PCR amplification of *NbCCTζ* gene. Lane1: PCR amplification of *NbCCTζ* gene; LaneM: DL2000 DNA ladder; Figure S2: The relative expression level of *NbCCTζ* of *N. bombycis*. The *NbCCTζ* expression levels at 96 h post-infection were used as reference. Error bars represent standard deviation of three independent replicates (*n* = 3, mean ± SE); Table S1: The summary of the expression pattern and subcellular localization of CCTζ, CCTδ and CCTα.
