# Supplementary material for: The Function of Different Subunits of the Molecular Chaperone CCT in the Microsporidium Nosema bombycis: NbCCTζ Interacts with NbCCTα
Source: J Fungi (Basel). 2024 Mar 20;10(3):229. doi: 10.3390/jof10030229 (PMC10971345; doi:10.3390/jof10030229)
Supplement: Supplementary file 1 [file jof-10-00229-s001.zip › Table s1.pdf]

Table S1. The summary of the expression pattern and subcellular localization of CCTζ, CCTδ and CCTα.

**Protein**	**the transcriptional level of the CCT subunit in *N. bombycis***			**The intracellular location distribution of CCT subunits**	
	Infective phase	Proliferative phases	Sporogonic phases	cytoplasm	nuclei
**CCTζ**	high	medium	low	+	-
**CCTδ**	high	medium	low	+	-
**CCTα**	low	medium	high	+	+
