# Supplementary figures and images for: The Function of Different Subunits of the Molecular Chaperone CCT in the Microsporidium Nosema bombycis: NbCCTζ Interacts with NbCCTα
Source: J Fungi (Basel). 2024 Mar 20;10(3):229. doi: 10.3390/jof10030229 (PMC10971345; doi:10.3390/jof10030229)

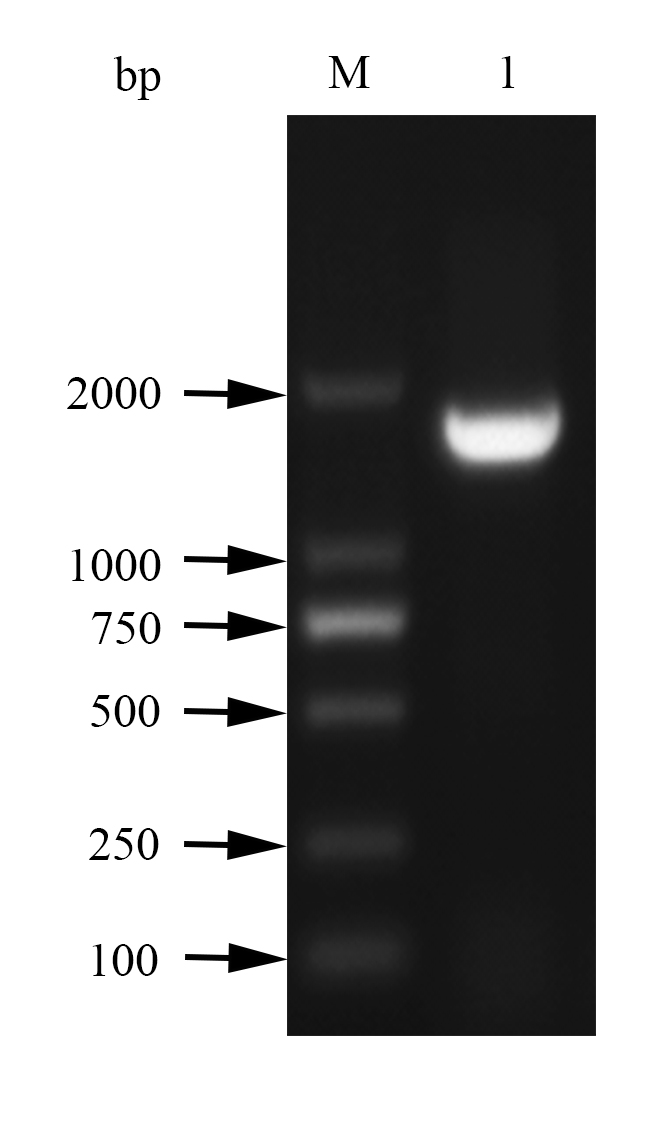

Supplement: Supplementary file 1 [file jof-10-00229-s001.zip › Figure S1.JPG]

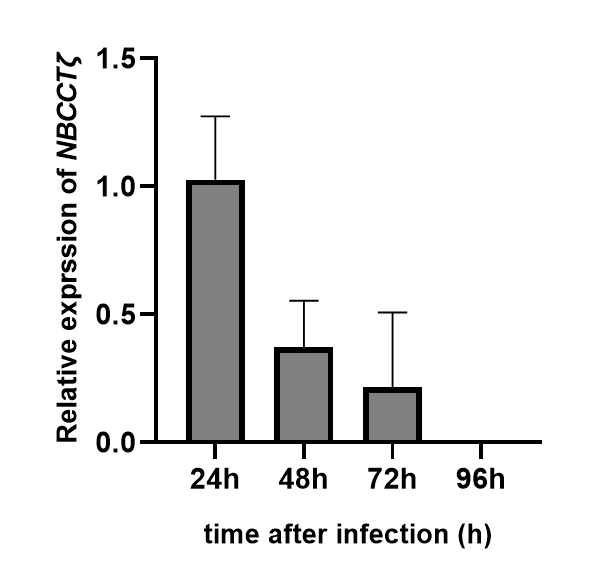

Supplement: Supplementary file 1 [file jof-10-00229-s001.zip › Figure S2.png]
